# Preclinical Toxicity Evaluation of Clinical Grade Placenta-Derived Decidua Stromal Cells

**DOI:** 10.3389/fimmu.2019.02685

**Published:** 2019-11-19

**Authors:** Behnam Sadeghi, Gianluca Moretti, Fabian Arnberg, Erik Samén, Bita Kohein, Rusan Catar, Julian Kamhieh-Milz, Sven Geissler, Guido Moll, Staffan Holmin, Olle Ringdén

**Affiliations:** ^1^Translational Cell Therapy Research (TCR), Department of Clinical Science Intervention and Technology (CLINTEC), Karolinska Institutet and Karolinska University Hospital, Stockholm, Sweden; ^2^Department of Clinical Neuroscience, Karolinska Institutet and Karolinska University Hospital, Stockholm, Sweden; ^3^Department of Neuroradiology, Karolinska Institutet and Karolinska University Hospital, Stockholm, Sweden; ^4^Department of Radiopharmacy, Karolinska Institutet and Karolinska University Hospital, Stockholm, Sweden; ^5^Department of Nephrology and Internal Intensive Care Medicine, Charité-Universitätsmedizin Berlin, Corporate Member of Freie-Universität Berlin, Humboldt-Universität zu Berlin, and Berlin Institute of Health (BIH), Berlin, Germany; ^6^Berlin Institute of Health (BIH), Berlin, Germany; ^7^Department of Transfusion Medicine, Charité-Universitätsmedizin Berlin, Corporate Member of Freie-Universität Berlin, Humboldt-Universität zu Berlin, and Berlin Institute of Health (BIH), Berlin, Germany; ^8^BIH Center for Regenerative Therapies (BCRT), Charité-Universitätsmedizin Berlin, Corporate Member of Freie-Universität Berlin, Humboldt-Universität zu Berlin, and Berlin Institute of Health (BIH), Berlin, Germany; ^9^Julius Wolff Institute (JWI), Charité-Universitätsmedizin Berlin, Corporate Member of Freie-Universität Berlin, Humboldt-Universität zu Berlin, and Berlin Institute of Health (BIH), Berlin, Germany; ^10^Berlin-Brandenburg School for Regenerative Therapies (BSRT), Charité-Universitätsmedizin Berlin, Corporate Member of Freie-Universität Berlin, Humboldt-Universität zu Berlin, and Berlin Institute of Health (BIH), Berlin, Germany

**Keywords:** placenta-derived decidua stromal cells, mesenchymal stromal cells, toxicity, side effects, cellular therapy

## Abstract

Placenta-derived decidua stromal cells (DSCs) are being investigated as an alternative to other sources of mesenchymal stromal cells (MSCs) for cellular therapy. DSCs are more effective in treating acute inflammatory diseases in human and this is our preclinical safety study of human DSCs in Sprague-Dawley rats and Balb/c mice. Human DSCs were cultured and expanded from fetal membranes obtained from placentas following cesarean section. In rats, 0.5 × 10^6^ cells/kg were injected intravenously (*n* = 4) or intra-aortal (*n* = 4). In mice, DSCs were given intravenously at doses ranging from 4–40 × 10^6^ cells/kg (total of *n* = 120 mice). *In vivo* tracking of human cells in mice was performed by using transduced DSC with luciferin gene, and in rats by using ^18^F-FDG PET. Clotting parameters were determined *in vitro* and *in vivo*. All intra-arterially DSC-treated rats had normal motility and behavior and histological examination was normal for liver, spleen kidneys and thigh muscles. Mice treated with DSCs showed no immediate or long-term side effects. None of the mice died or showed acute toxicity or adverse reactions 3 and 30 days after DSC infusion. Murine blood biochemistry profiles related to liver, kidney, heart, and inflammatory indices was not influenced by DSC infusion and complete blood counts were normal. *In vivo* tracking of infused DSCs detected a signal in the lungs for up to 4 days post infusion. Compared to bone marrow derived MSCs, the DSCs had better viability, smaller size, but stronger clotting in human blood and plasma. Both MSC- and DSC-induced coagulation and complement activation markers, thrombin-anti-thrombin complex (TAT) and C3a, and *in vitro* clotting parameters were decreased by heparin supplementation. In conclusion, DSCs are safe with almost no side effects even with doses 40 times higher than are used clinically, particularly when supplemented with low-dose heparin.

**Graphical Abstract:**
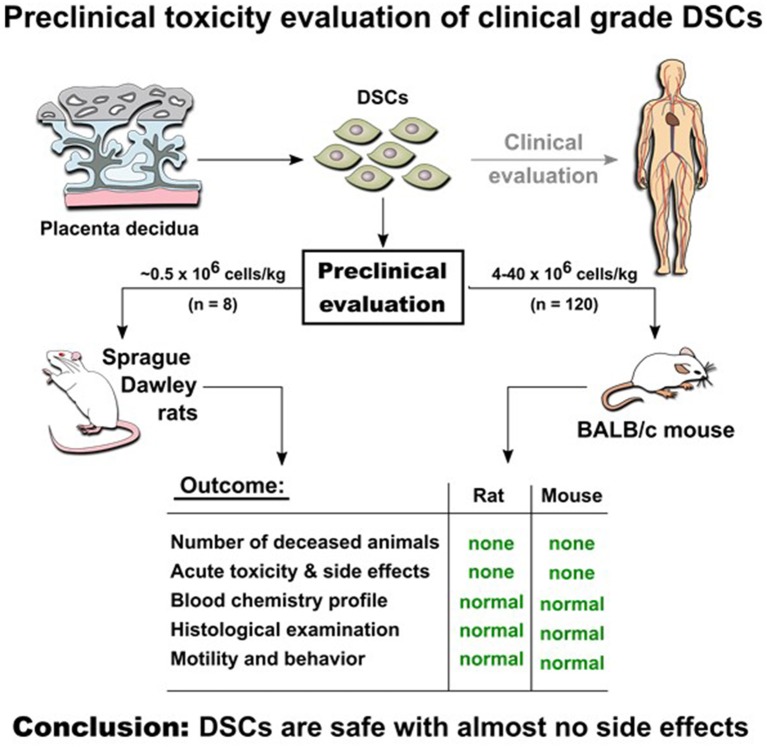
Experimental *in vivo* studies indicate safety of DSCs infusion in two animal models.

## Introduction

Mesenchymal stromal cells (MSCs), first described by Friedenstein et al. (1), have the potential to differentiate into several mesenchymal lineages and are found in many vascularized human tissues (2, 3). MSCs have multiple beneficial properties; e.g., they support hematopoiesis and have potent immunomodulatory property, and have therefore been in experimental clinical use for treatment of a series of inflammatory diseases, including graft-vs.-host disease (GvHD) and hemorrhagic cystitis following hematopoietic stem cell transplantation (HSCT), autoimmune diseases and in regenerative medicine (4–10). Galleu et al. demonstrated that infused MSCs are actively induced to undergo perforin-dependent apoptosis by recipient cytotoxic cells (11) and this process appears to be required for MSC-induced immune suppression (8, 12–14). Galipeau and Sensébé reasoned that the clearance of apoptotic MSC-like cells and in particular lung-embolized placental stromal material leads to reprograming of lung macrophages by efferocytosis, thus promoting fetomaternal tolerance (8).

Infusions of placenta-derived decidual stromal cells (DSCs) may thus mimic a highly conserved biological process in mammals that induces systemic immunomodulation and feto-maternal tolerance during pregnancy (8, 15–17). Placental DSCs differ from bone marrow (BM)-MSCs in several aspects. Compared to MSCs, the DSCs are only half the size, show less differentiation into chondrocytes and osteocytes, have a stronger inhibitory effect on allo-reactive T-cells, and promote stronger coagulation (18–20).

Systemic or local administration of clinical grade MSCs derived from various adult and perinatal tissue sources have been used in both the autologous and allogeneic transplantation setting for many decades (21). Numerous preclinical and clinical studies have evaluated the safety and side effects of therapeutic MSCs (15, 22–24). Nonetheless, some reports on potential adverse events highlight a general need for better MSC characterization and handling (15, 24, 25). Multiple research and clinical groups recently reported that heparin improves both the safety and efficacy of MSC therapy (18, 26, 27).

Our initial two clinical reports showed that intravenous infusion of human BM-MSCs and DSCs triggers an innate immune attack, termed the instant blood-mediated inflammatory reaction (IBMIR) (15, 18, 28). Liao et al. recently confirmed this finding demonstrating that BM-MSCs are not fully compatible with blood due to their intrinsic Tissue Factor (TF/CD142) expression, particularly after extensive expansion, which was furthermore found to be conserved among different species of mammals (27).

Liao et al. found that large doses of MSCs induced symptoms of respiratory and/or heart failure attributed to the triggering of intravascular thrombosis promoting cell embolization in the lungs (27). In contrast, clinically more relevant MSC doses induced only mild and reversible coagulation, but anticoagulation with heparin (400 U/kg) effectively prevented MSC-induced coagulation and concomitant adverse events of large cell doses.

The most common cell dose infused in patients is 1–2 × 10^6^ cells/kg, but does up to 10–20 × 10^6^ cells/kg have also been tested (15). Thus, a major bottleneck is the need for robust *ex vivo* expansion of GMP grade cell product to generate clinically relevant cell doses (25). A practical solution to overcome these restrictions may be the use of MSCs generated from other tissue sources with a more favorable amount of starting material and better growth characteristics during *ex vivo* expansion, such as placenta-derived DSCs.

We previously reported on the good safety and efficacy of DSCs in treatment of GvHD and HC following HSCT (29, 30) as well as in experimental setting (31, 32). When employed at the typical low clinical cell doses, DSCs demonstrated a safe toxicity profile and no side effects in the clinical setting (33) and in an animal model of BMT and GvHD (31). This is in analogy to very recent reports by Perlee et al. that the infusion of clinical-grade TF-bearing adipose tissue (AT)-MSCs is safe at the typically employed clinical doses (34, 35).

We here report the comprehensive preclinical toxicity study of systemic DSC infusion in two animal models (rat and mouse) and in series of *in vitro* assays employing human blood.

## Materials and Methods

### Isolation, Quality Testing, and Reconstitution of Stromal Cells

The BM-MSCs and placenta DSCs were isolated and characterized as described previously (18, 29). The MSCs were obtained from BM aspirates of adult healthy volunteer donors and the placentas were collected following elective cesarean section delivery after obtaining informed consent. Donors were negative for HIV, hepatitis B and C infection and syphilis. The cell culture was done in clean rooms and culture media and cell suspensions were negative for bacteria and fungi and polymerase chain reaction (PCR)-negative for Mycoplasma. The cells were expanded for up to four passages in a medium containing 10% fetal calf serum (FCS; Hyclone, Logan, UT).

Flow cytometric analysis was conducted on cells labeled with the antibodies outlined in supporting information, Table S1. The cells were labeled with antibodies, fixed with 1% paraformaldehyde, and analyzed on a FACS Aria (Becton Dickinson, Franklin Lakes, NJ); 5,000 gated events were quantified and analyzed with Summit v.4.1 (Dako, Glostrup, Denmark) as previously described (36). The adipogenic and the osteogenic differentiation capacities were evaluated as described previously (37). DSCs did not differentiate well to bone and fat in contrast to BM-MSCs as previously reported in detail (19). DSCs were positive for typical MSC markers CD105, CD166, CD73, CD90, and CD29. They were negative for hematopoietic markers CD34, CD45, and CD14. A detailed FACS analysis of DSCs was previously published (37).

The cells were stored in liquid nitrogen and thawed for intravenous infusion or the *in vitro* assays and washed twice in buffer containing 5% human serum albumin (HSA; CSL Behring, Marburg, Germany). Cell viability, cell size, and other morphological parameter of the reconstituted products were assessed by trypan blue exclusion dye, flow cytometry, and automated electrical impedance-based Cell Counter (CASY-TT; Roche, Germany), as previously described (18, 36).

### Intra-Arterial/Venous Infusion Toxicity Evaluation in Rats

All animal experiments were conducted with ethical approval given by the local authority (Stockholms Norra djurförsöksetiska nämnd, ethical approval #N138/10). Adult male Sprague-Dawley rats (mean body weight = 340 ± 4.9 grams) were permitted to have access food and water *ad libitum* until surgery. Anesthesia was induced using 4% isoflurane mixed with 100% O_2_ and subsequently maintained at 2% isoflurane. The animals were kept normothermic by means of a rectal thermistor coupled with a heating pad. For intravenous cell administration the tail vein was cannulated (*n* = 4).

For intra-aortal administration a midline incision (5 mm) was made proximally on the ventral side of the tail (*n* = 4). The fascia covering the ventral artery was cut, and the exposed artery was ligated distally. Next, a 7-0 silk ligature was tied loosely around the proximal part of the exposed artery, and a microvascular clip was placed over the ventral artery. The artery was cut and a “0.0157” “Sonic” hydrophilic micro-catheter (Balt Extrusion, France) carrying a micro wire was advanced to a tip position in the thoracic aorta With the catheter in this position, cell suspensions containing 0.5 × 10^6^ DSC dispersed in 0.5 ml saline with 10% fetal calf serum were infused during 1 min. After infusion, the micro-catheter was retracted and the proximal ligature on the tail artery was tightened and the incision was closed.

The animals were returned to their cages with food and water *ad libitum*. The animals were weighed before and 24 h after cell injection. Activity, gait, grooming and motility were evaluated at 2, 4, and 24 h for gross evaluation of the animals' conditions.

### Histopathology Evaluation of Rat Organs

Twenty four hours after the transplantation, all animals were sacrificed by decapitation under identical anesthesia as during the initial surgery. The left kidney, liver, spleen and left thigh muscles were snap-frozen and stored at −80°C. Next, 14 μm sagittal cryo-sections were cut serially throughout the organs and stored at −20°C. The sections were rehydrated, fixed in formaldehyde and stained with Hematoxylin-Eosin according to Mayer's protocol. Microscopical analysis was performed on a sample of 10 sections at even intervals throughout the organs to assess presence of tissue infarction.

### Short- and Long-Term Toxicity Assay Following DSC Administration in Mice

Female Balb/c mice, 10 to 12 weeks old, were purchased from Scanbur (Sollentuna). The mice were maintained under pathogen-free conditions with controlled humidity (55% ± 5%), 12 h of alternating light and dark, controlled temperature (21 ± 2°C) and high efficiency particulate air (HEPA)-filtered air. Groups of 5–10 mice were kept in individually ventilated cages and were fed autoclaved mouse chow and tap water *ad libitum*. The South Stockholm Ethics Committee for Animal Research approved all the experiments described here (No 8/16).

The DSCs were thawed and washed as outlined above, passed through a 70 μm cell strainer and suspended in sodium chloride 0.9% containing 5% HSA (infusion buffer). Four different doses of DSC ranging from 0.1–2–10^6^ cells/mouse were reconstituted in 200 μl of infusion buffer. In some experiments to evaluate the effect of anticoagulation, 10 Units/animal of heparin were added to the infusion buffer. In the toxicity study each mouse received a single infusion of DSC via a lateral tail vein. The mice were humanly killed 3 or 30 days after cell infusion.

### Blood Analysis and Serum Biochemistry Analysis in Mice

At designated time (2, 8, 24 h, +3 and +30 days after cell infusion) and after giving anesthesia to the animals, peripheral blood was collected in different type of tubes (EDTA, citrate or heparin tubes). Blood samples were centrifuged immediately and serum or plasma was transferred into new tubes and placed in −80°C until analysis. Frozen serum or plasma was transported with dry ice to the research section of the central blood- and biochemistry lab at the Karolinska University Hospital. Different biochemical indices were analyzed using various instruments according to the manufacturer instruction. Final data was sent back to us in Excel files. Formation of the blood activation markers thrombin-anti-thrombin complex (TAT) and complement component C3 activation fragment a (C3a) in murine plasma was measured with ELISA (Cusabio Biotech LTD, Wuhan, China), at 2, 8, and 24 h after the DSC infusion.

### DSC Labeling and *in vivo* Cell Tracking in Mice and Rats

Transfection and transduction of DSCs with luciferin gene containing green fluorescent protein (GFP), was performed as explained elsewhere (38). Lentiviral vector co-expressed GFP and luciferin was kindly provided by Joseph C. Wu (University of California San Francisco, Institute for Regenerative Medicine). Briefly, the virus supernatant was harvested 24 and 48 h after transfection and concentrated by centrifugation at 6,000 g for 16 h at 4°C. The DSCs were infected with the virus supernatant overnight in the presence of polybrene (8 mg/mL). Transduced cells were selected by adding puromycin (1.5 mg/mL) for 48 h Transduced. GFP+ cells were confirmed by use of fluorescence microscopy. Transfected DSCs were sorted using flow cytometry and GFP+ cells were isolated. The purity of GFP+ cell was more than 99%. The surface markers expression profile of DSC-Lu+ was tested with flow cytometry. The DSC-Lu+ immunomodulatory function was evaluated using the mixed lymphocyte reaction (MLR) assays (31).

For the *in vivo* bio-distribution study, a group of Balb/c mice received 1 × 10^6^ DSC-Lu+ cell via a lateral tail vein. At different time points, +1, +4, +6 h, and 1, 2, 3, 4, and 7 days after cell infusions the mice were imaged using a Xenogen IVIS100 imaging system, using the manufacturer's directions. For *in vivo* imaging of the cells, mice were anesthetized with isoflurane and injected intraperitoneally with 50 mg/kg D-luciferin (Caliper, Hopkintown, MA) at 5–10 min was given before imaging. To control the background photon emission, the obtained data were subjected to average background subtraction, using data from control animals that were only injected with identical doses of luciferin.

For *in vivo* bio-distribution studies in rats, Sprague-Dawley rats were anesthetized and catheterized as described above. Next, the animals received intra-aortal injections (*n* = 2) and tail-vein injections (*n* = 2) with 1 × 10^6^ DSCs labeled with [2-^18^F]-2-fluoro-2-deoxy-D-glucose (^18^F-FDG) for dynamic *in-vivo* positron emission tomography of cell trafficking immediately after injection. The ^18^F-FDG used was an aliquot obtained from daily productions for clinical PET at the Karolinska University Hospital and had passed all quality requirements for administrations in humans. ^18^F-FDG (10–20 MBq, 500 μL) followed by a saline flush of 300 μL.

### Histopathology Evaluation of Organs in Mice

Sample preparation was conducted as explained previously (39). Briefly, at the appropriate time point (day +3 and +30 after cell infusion), mice (*N* = 6 in each group/time point) were given general anesthesia and killed by cervical dislocation. Lung, liver, kidney, spleen, and GI tract were harvested and placed in a sealed container with 4% paraformaldehyde. After harvesting, the lungs were perfused with 4% paraformaldehyde through the trachea, and then transferred to the sealed container. After 24 h the tissues were transferred to 70% ethanol and were thereafter dehydrated using increasing concentrations of ethanol, cleared with xylene and embedded in paraffin. The tissues were cut 5 μm thicknesses and deparaffinized with tissue clear reagent, rehydrated in series of ethanol in decreased concentrations. The sections were stained with hematoxylin and eosin, dehydrated in ethanol. Tissue Tek Prisma (Sakura Finetek Inc., Torrance, CA, USA). An automated slide-stainer was used for the staining procedure according to the manufacturer's recommendations. Samples from different treatment as well as the control group were observed and scored by a blinded animal histopathologist.

### Clotting Analysis of DSCs in Human Blood and Plasma *in vitro*

The clotting time (in seconds) was recorded on a semi-automatic 10-channel ball coagulometer (MC10plus; Merlin Medical ABW Medizin und Technik GmbH, Lemgo, Germany), as reported earlier. Frozen aliquots of DSCs were thawed, washed twice, and re-suspended in buffer containing 10% HSA, with or without supplementation of low-dose heparin, as indicated in the figure legends. Sodium citrate anti-coagulated human blood was obtained from healthy volunteers who had not received any medication for at least 10 days, and citrated plasma was collected after centrifugation at 1,000 × *g* for 10 min. The cuvette was filled with either 100 μl of citrated blood diluted 1:1 in PBS, or 100 μl of citrated normal plasma. Blood or plasma was then supplemented with 50 μl of buffer with or without 3,000 stromal cells to a final concentration of 15,000 cells/ml corresponding to a dose of 1–2 × 10^6^ cells/kg as typically used in clinical trials. To initiate clotting, 50 μl of 40 mM Ca^2+^ solution was added, to a final concentration of 10 mM.

### Statistical Analysis

All data are expressed as mean ± standard error (SE) unless otherwise stated. Differences between groups were analyzed by ANOVA and the Student's *t*-test was used. If the data did not fit a normal distribution, the Mann-Whitney test or the Wilcoxon matched-pairs test was used (two-tailed, 95% confidence intervals). Any *P* < 0.05 were considered statistically significant. Prism software 5.0 was used for analysis and making the graphs (GraphPad Software, La Jolla, CA).

## Results

### The Effect of IA/IV Infusion of DSC in Rat and Biodistribution and Safety Evaluation Following Intra-Arterial DSC Infusion

In a first exploratory experiment in rats, a dose of 0.5 × 10^6^ cells/animal was slowly infused either by cannulation of the tail-vein or by assistance of a micro-catheter into the abdominal aorta to assess principal DSC toxicity. All DSC-treated rats were healthy and no short time adverse effects were observed.

No negative effect on body weight was seen in any of the animals at 24 h. All animals showed normal motility and exploratory behavior at 2, 4, and 24 h post cell infusion. Macroscopic and histological analysis of internal organs did not reveal any evidence for infarction, hemorrhage or pathologic lesion (data not shown).

**Figure 1:**
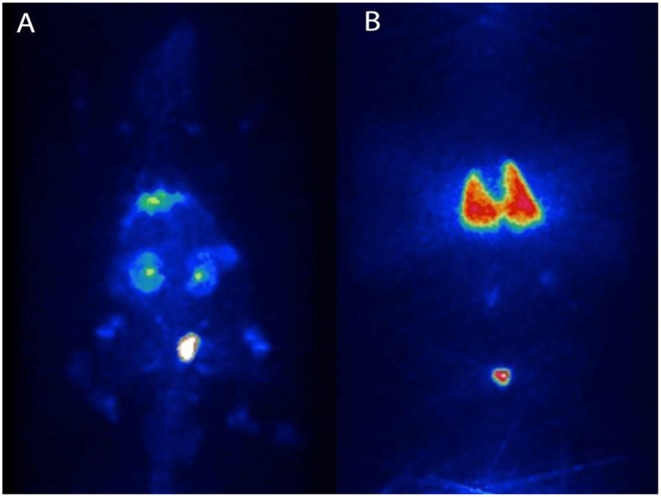
Migration pattern of i.a and i.v. infused human DSCs in the rat. Naïve Sprague-Dawley rats received intra-aortal (i.a) injections (*n* = 2) and tail-vein (i.v) injections (*n* = 2) with 1 × 10^6^ DSCs labeled with [2-^18^F]-2-fluoro-2-deoxy-D-glucose (^18^F-FDG). Dynamic *in-vivo* positron emission tomography of cell trafficking was done immediately after injection. **(A,B)**, are cell distribution patterns following i.a and i.v infusion of DSC, respectively.

Injections of ^18^F-FDG-labeled DSCs and micro-PET imaging 90 min following DSC i.a. injection in rats showed radioactivity distribution primarily in the abdominal organs whereas i.v. infusion resulted in radioactivity distribution almost exclusively in the lungs (Figure 1).

To examine possible effects on organs if the DSCs were administered intra-arterially, we performed intra-aortic infusions of cells proximal to the renal arteries by endovascular technique and analyzed clinical behavior and possible ischemic events in the liver, kidney, spleen and muscles in the lower extremities. During the 24 h follow up, the animals did not lose weight, showed normal activity, gait, grooming and motility. H&E staining of liver, kidney, spleen, and muscle sample were normal and without evidence of ischemic- or other tissue injury (data not shown).

### Short- and Long-Term Toxicity in Mice

All animals tolerated the intravenous cell infusion even by the dose of 1 × 10^6^ cells/mouse (*N* = 5–9 in each group/time point), which is equal to 40 × 10^6^ cell/kg. None of them showed any immediate side effects including restless, breathing problem (dyspnea) or any changes in grooming and activity.

**Figure 2:**
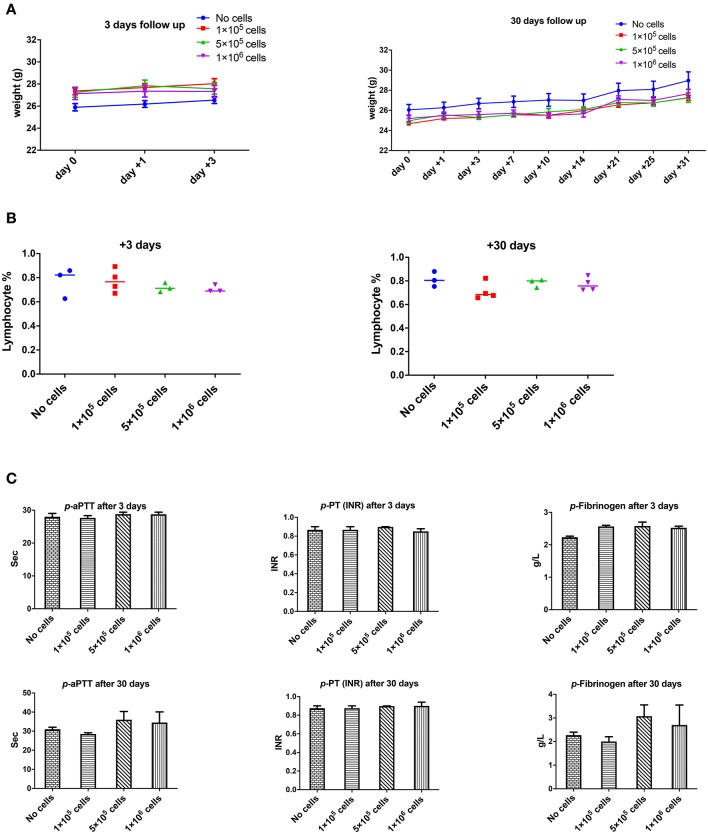
The effect of human DSCs infusion on the mice weight, blood and coagulation system. To evaluate the clinical and biological changes in animals, human DSCs were infused i.v. at different doses (0–1 × 10^6^ cell/mouse). The outcome was measured in short (+3 days after) or long term (+30 days after) follow up (*N* = 3–5 mouse per dose/timepoint). **(A)** Weight and general health of treated animals was not affected by any dose in short and long term. **(B)** The frequency of peripheral blood lymphocyte did not change after human cell infusion. **(C)** Plasma level of coagulation factors was not influenced by the cell infusion as well.

However, the dose of 2 × 10^6^ cell/mouse (equal to 80 × 10^6^ cells/kg) should be infused slowly and carefully otherwise rapid infusion might induce restless, dyspnea and finally death (2 out of 5 animals). It seems that the problem could be due to transient lung emboli (observed in few numbers of healthy mice), as observed by Perlee et al. in their mouse model (35), but which resolved within 24 h. No changes in body weight were seen in any of the mice 3 or 30 days after DSC infusion (Figure 2A).

### The Effect of DSC Infusion on Blood Count and Coagulation

None of the complete blood count (CBC) items changed in short- or long-term follow-up after different doses of intravenous DSC infusion in mice. Peripheral blood lymphocyte frequency did not alter by cell dose at different time points (Figure 2B).

One of the most important issues with intravascular cell therapy procedures is the evaluation of coagulopathy and the blood clotting system. We evaluated the effect of DSC infusion on the mice coagulation system. As shown in Figure 2C, coagulation cascade proteins or the protease was not affected by the DSC infusion and different doses of DSCs did not change activated partial thromboplastin time (aPTT), prothrombin time/international normalized ratio (PT-INR) and Fibrinogen level neither in short nor long term.

### Plasma Inflammatory and Hemolysis Markers and DSC Infusion

**Figure 3:**
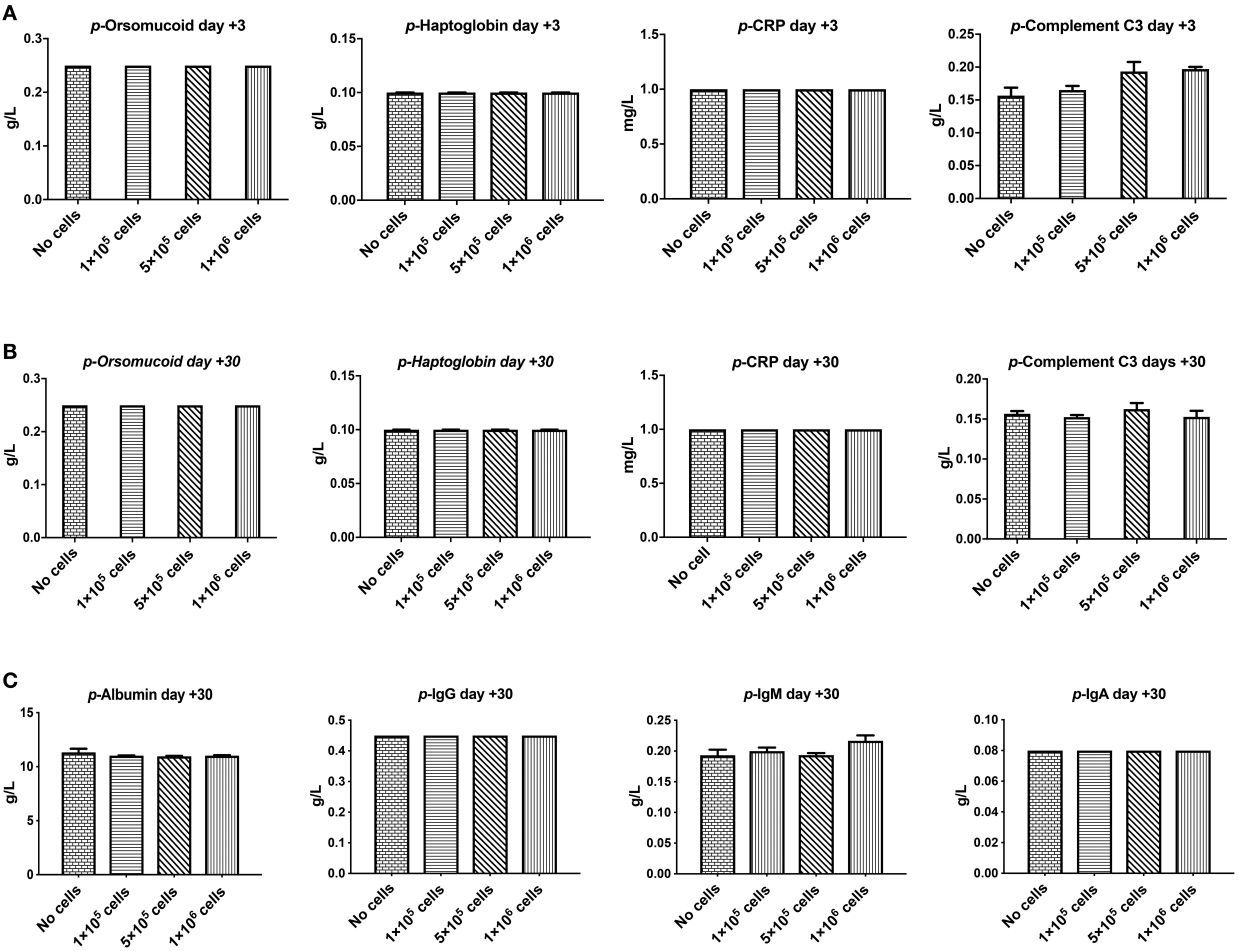
The effect of human DSCs infusion on the inflammatory markers, serum proteins level. Early and delayed inflammatory reaction could be an important and relevant side effect following systemic cell therapy. Healthy mice received human DSCs i.v. at different doses (0–1 × 10^6^ cell/mouse) (*N* = 3–5 mouse per dose/timepoint). **(A–C)** The acute inflammatory reactions' protein as well as serum level of immunoglobulin and albumin were measured and showed no significant changes in the short (+3 days after) or the long term (+30 days after) follow up.

Orosomucoid, Haptoglobin, and C-reactive protein that are indicators for acute inflammation response and hemolysis were evaluated at 3 and 30 days after different doses of DSC infusion in mice. As shown in Figures 3A,B none of these indices were changed following cell infusion. The major plasma complement C3 levels did not show any significant deviation after different cell dose infusions (Figure 3B).

We also evaluated other plasma proteins especially immunoglobulin and albumin levels. DSC infusion did not induce any changes in plasma protein levels, and it seemed that up to 1 × 10^6^ cells/mouse are safe for plasma protein balance both in the short and in the long term follow up (Figure 3C).

### Liver and Kidney Function Was Not Affected by DSC Infusion

**Figure 4:**
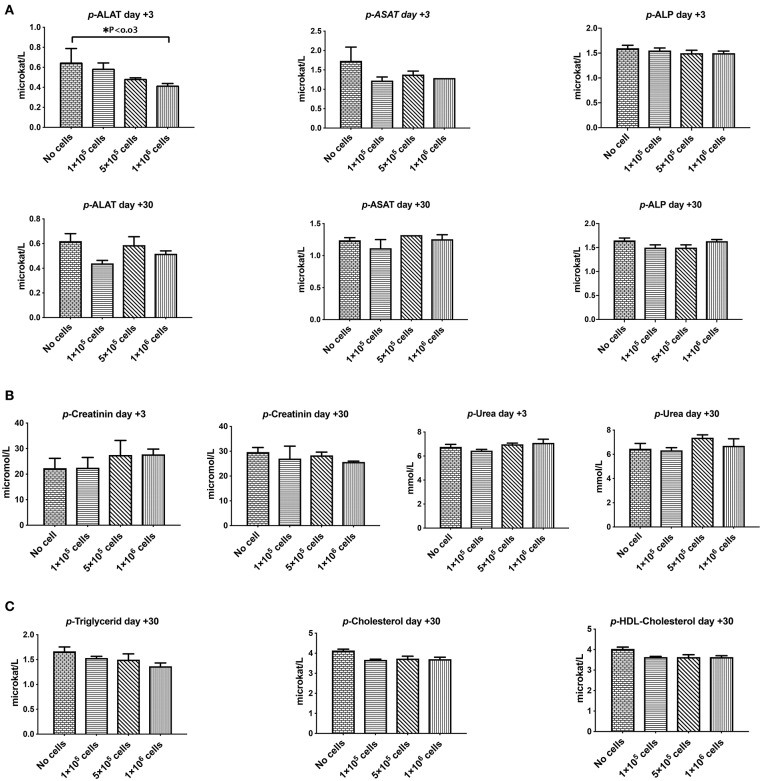
The effect of human DSCs infusion on the liver and kidney function. Organ toxicity is one of the most important concerns in all cell based therapy protocols. Healthy mice received human DSCs i.v. at different doses (0–1 × 10^6^ cell/mouse) (*N* = 3–5 mouse per dose/timepoint). **(A)** Liver enzymes **(B)** kidney function biomarkers and **(C)** serum lipid profile were measured and compared among different group in short and long term following cell infusion. Interestingly, human cell infusion ranged from 0–40 × 10^6^ cell/kg and did not show any toxicity relevant to these organs.

Liver and kidney functions tests are crucial toxicity evaluation for any new medication. As shown in Figure 4, plasma levels of liver parameters ALAT, ASAT, ALk P as well as creatinine and Urea were not affected by any cell dose at short or long-term assays (Figures 4A,B). On the contrary, a high dose of DSCs infusion significantly decreased the ALAT level in manipulated animals. DSC infusion did not affect the plasma lipid profile, although some non-significant decrement in the plasma levels of cholesterol and triglyceride were observed at day +30 (Figure 4C).

### *In vivo* Tracking of the Infused Therapeutic Cell Products in Mice

**Figure 5:**
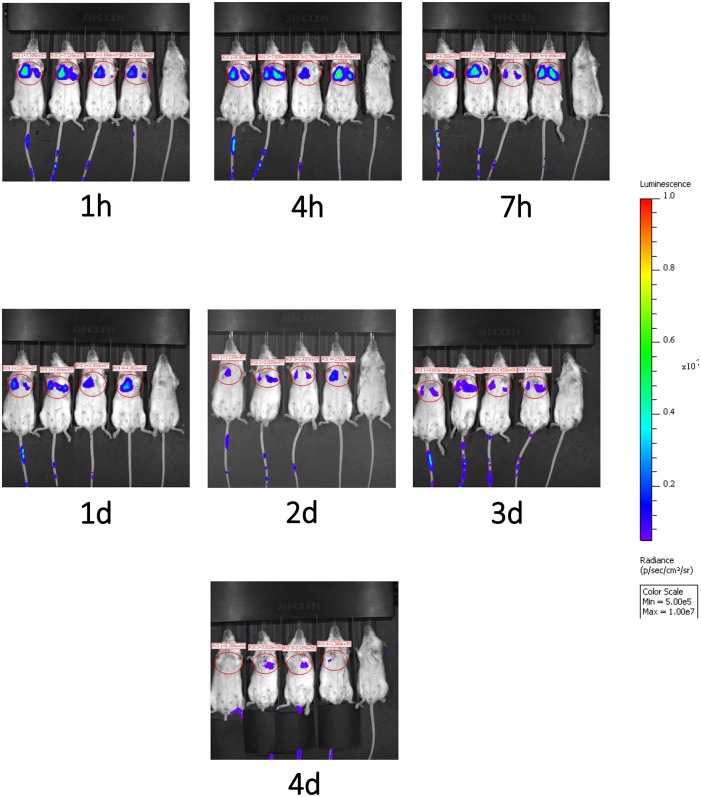
Migration pattern of i.v. infused human DSCs in healthy mice. Migration pattern of systemic infused stromal cells, in live animals, was done by using labeled cells. Human DSCs were transduced with Luciferin gene containing GFP using lentivirus vector (methods). 1 × 10^6^ cell/mouse were infused i.v. and after light anesthesia, live animals (*N* = 2) were kept in IVIS CCD camera and at different time points whole body scan were recorded. Infused cells first pass to the lung and stayed there up to 4 days. In the Luciferin tracking model, just live cells will be recorded. It means that the human DCs mainly reside it the lung.

We next evaluated the migration pattern of injected cells in healthy animals. The infused DSC first moved to the lung (Figure 5), and the signal continuously faded with time. Signals in the lungs were detected up to 4 days after cell infusion, but not thereafter. We could not detect any significant signals in liver or spleen at any evaluated time period (Figure 5).

### Histopathology Evaluation of Liver, Kidney, and Lung Following DSC Infusion

**Figure 6:**
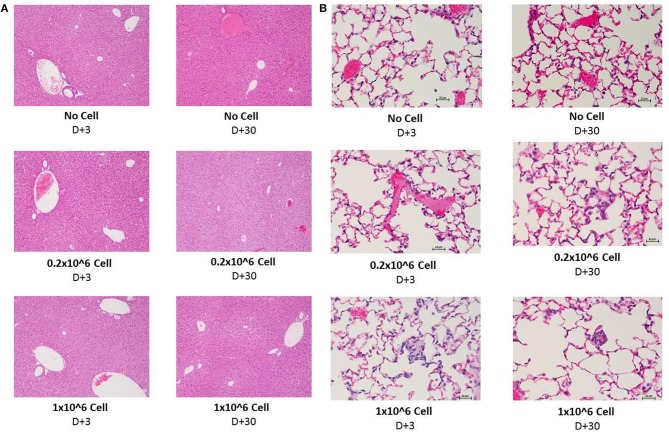
Histopathology evaluation of lung and liver after i.v. infusion of human DSCs in healthy mice. To assess the effect of cell infusion on vital organs liver, lung and kidney were prepared for histopathology evaluation 3 and 30 days after different doses of cell infusion (*N* = 3 mouse per dose/timepoint). **(A)** Liver tissue with no cell, 0, 2, and 1 × 10^6^ DSC IV injection (+heparin) 3 days (left column) and 30 days after cell infusion. **(B)** Lung tissue with no cell, 0, 2, and 1 × 10^6^ DSC IV injection (+heparin) 3 days (left column) and 30 days after cell infusion.

Histopathology evaluation of the liver at short (+3) or long term (+30) follow up did not show any significant abnormality related to cell infusion. Mild diffuse glycogenesis, occasional individual cell with micro vesicular lipidosis, moderate cell vacuolization; moderate karyomegaly/anisokaryosis; scattered gray-green pigment in kupffer cells among others were observed in liver samples. However, none of this observations were related to cell infusion or even cell dose. Heparin infusion did have any effect on histopathological manifestation (Figure 6A). Kidney tissue was not essentially affected by any dose of cell infusion with or without heparin at any time point (data not shown).

The lungs are the first organs in which the infused cells will arrest. Thus, histopathology signs for thromboembolic features, hemorrhage and granulomatous formation were carefully evaluated among different groups. DSC infusion did not induce any of the mentioned pathologic features in the short term (+3) period. Heparin also did not change the histopathology pattern in short term.

Moderate diffuse hyperemia; moderately frequent interstitial foci of unidentified/inflammatory cells; scattered foamy macrophages and occasional megakaryocytes were seen more often in the 30 days follow up. Few vessels contain apparently coagulated material. However, it did not have any correlation to cell dose or heparin treatment. This finding was also observed in the control group (Figure 6B).

### Cell Morphology, Immunophenotype, and *in vitro* Clotting Analysis of Stromal Cells With and Without Addition of Heparin

**Figure 7:**
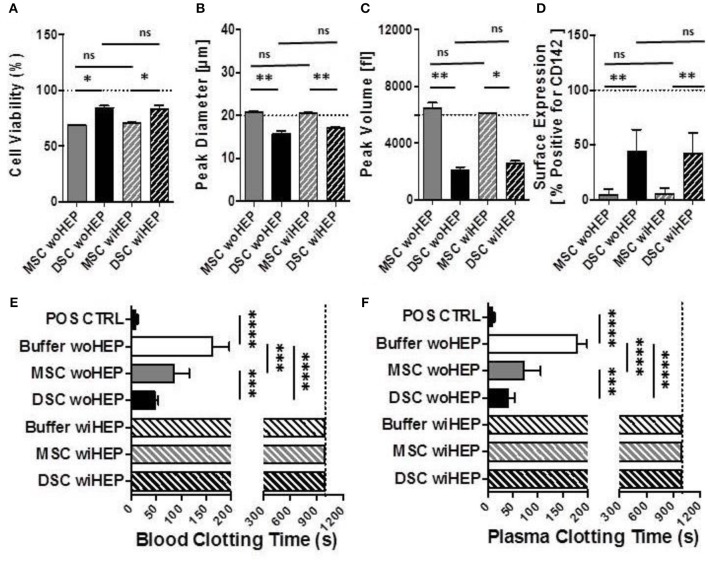
Cell morphology, immunophenotype, and *in vitro* clotting analysis of stromal cells prepared with and without addition of heparin. In order to simulate the preparation and formulation of our clinical products, aliquots of cryobanked therapeutic bone marrow mesenchymal stromal cells (MSC) and placenta-derived decidua stromal cells (DSC) were thawed, reconstituted and supplemented in buffer supplemented with or without low-dose heparin (HEP) as commonly done during clinical procedures, and then evaluated for viability, phenotype, and clotting time in human blood and plasma. **(A–C)** Cell suspensions of stromal cell batches from different donors (*n* = 4 each cell type) were analyzed with automated electrical impedance-based CASY counter for quantification of parameters of importance for systemic cell infusion: **(A)** Cell viability (%), **(B)** Peak diameter (um: micrometers), and **(C)** Peak volume (fL: femtoliters), **(D)** Flow cytometric analysis for cell surface expression of Tissue Factor (CD142, % of cells positive, *n* = 8) compared to isotype control, and **(E,F)** Analysis of clotting time (in seconds, *n* = 14 tests) after exposure of stromal cells (15,000 cells/mL) resuspended with and without heparin (10 U/mL) to fresh recalcified human blood **(E)** or plasma **(F)**, using a semiautomatic 10-channel ball coagulometer. Mean ± SD. ^*^P < 0.05, ^**^P < 0.01, ^***^P < 0.001, and ^****^P < 0.0001, and ns, not significant.

Compared to BM-MSCs, the DSCs had 16% better viability post thawing (Mean 69% vs. 85%, *P* = 0.05, Figure 7A). The DSCs had a smaller size than MSCs (Mean peak diameter 16 vs. 20 μm, *P* = 0.05, Figure 7B) and a smaller cell volume (Mean peak volume 2,000 vs. 6,500 fl, *P* < 0.05, Figure 7C). CD142 expression was higher for DSCs than BM-MSCs (Mean 43% vs. 5% positive, *P* < 0.001, Figure 7D). All parameters were unchanged by the addition of heparin (Figures 7A–D).

There was a stronger clotting of DSCs compared to BM-MSCs in human blood and plasma *in vitro* (*P* < 0.001, Figures 7E,F). Clotting was stronger for DSCs (75% reduction) than MSCs (50% reduction) when compared to the buffer controls (*P* < 0.0001 and *P* < 0.001, respectively). Clotting in human blood and plasma was abrogated for both cell types by the addition of low-dose heparin (Figures 7E,F).

### *In vivo* Monitoring of Systemic Blood Parameters After Stromal Cell Infusion With and Without Heparin in Mice

**Figure 8:**
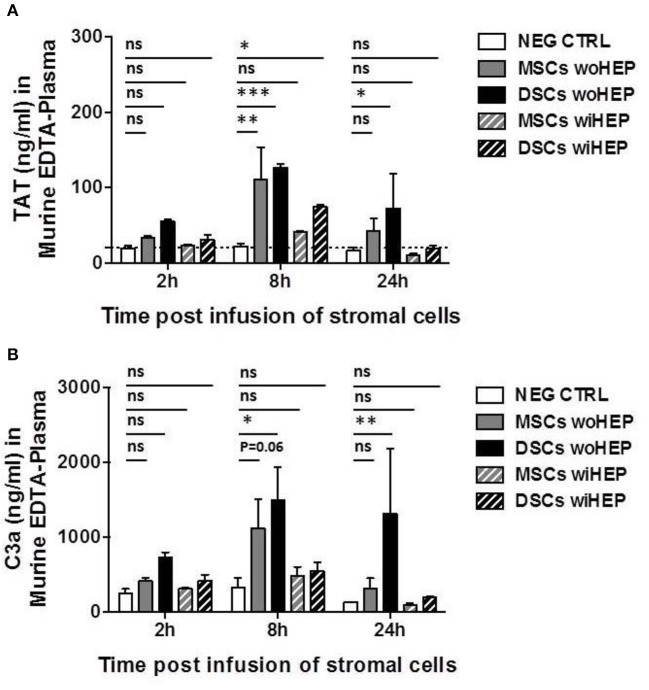
Analysis of systemic coagulation and complement activation markers in mice after systemic infusion of stromal cells prepared with and without heparin. Aliquots of cryobanked therapeutic bone marrow mesenchymal stromal cells (MSCs) and placenta-derived decidua stromal cells (DSCs) were thawed, washed and reconstituted in buffer supplemented with or without low-dose heparin (HEP, final dose 10 units/animal), and infused at 500,000 cells/animal (*n* = 2 animals each bar), and kinetics of coagulation and complement activation markers monitored after 2, 8, and 24 h. Quantification of: **(A)** Coagulation activation marker TAT (ng/mL) and **(B)** Complement activation marker C3a (ng/mL) in murine EDTA-plasma. Mean ± SD. ^*^P < 0.05, ^**^P < 0.01, and ^***^P < 0.001, and ns, not significant. Dashed lines indicate background levels of C3a and TAT. TAT, thrombin-anti-thrombin complex, and C3a, complement component 3 activation fragment a.

The peak for the generation of coagulation activation marker TAT was found at 8 h post BM-MSC and DSC infusion compared to the buffer control (*P* < 0.01 and *P* < 0.001, respectively, Figure 8A). TAT was still detected following DSCs, but not following BM-MSCs infusion after 24 h (*P* < 0.05). TAT generation was decreased by the supplementation of heparin (10 U/animal), with minor TAT formation at 8 h post DSCs infusion (*P* < 0.05) (Figure 8A).

Similarly, complement activation marker C3a demonstrated a peak at 8 h post BM-MSC and DSC infusion compared to the buffer control (*P* = 0.06 and *P* < 0.05, respectively, Figure 8B). This was still evident for DSCs but not BM-MSCs at 24 h post cell infusion (*P* < 0.01), C3a generation was antagonized by heparin, with only weak and non-significant C3a formation at the observed time points (Figure 8B).

## Discussion

There is a bulk of data regarding the safety of BM-MSCs (22, 40). All showed that BM-MSCs are safe to infuse with few if any side effects (15). DSCs differ in many ways from stromal cells from other tissues such as bone marrow (18, 19). Therefore, comprehensive safety analysis is needed for DSCs.

Before treating patients, we injected DSCs to rats, as reported in this article and in rabbits (32) and found it to be safe with no side effects. Subsequently, we treated patients with acute and chronic GvHD, hemorrhagic cystitis and acute respiratory distress syndrome (30, 33, 41, 42). Side effects were minimal and severe adverse events were those commonly seen in patients undergoing allogeneic HSCT, such as infections, hemorrhages, graft failure and multi-organ failure (29, 33).

DSCs like other cells first go to the lung after i.v. infusion (41, 43, 44). These findings are confirmed in mice and rats in the present study, where homing to the lungs is seen up to several days after i.v. infusion. Special attention should be made to evaluate possible DSCs induced side-effects in the lungs such as thrombosis or pneumonias. We found no pulmonary embolism in the present study where DSC were infused to mice in 40 times higher dose than used in humans, which is in agreement with studies on AT-MSCs by Perlee et al. (34, 35).

We previously reported that mice, treated with total body irradiation prior to high dose DSCs infusion (20 × 10^6^/kg), died from pulmonary embolism (31). Using BM-MSCs, an increased risk for pneumonia-related death after HSCT was found at our center (45). DSCs have a stronger effect on coagulation compared to BM-MSCs (18). However, DSCs are only half the size of bone marrow MSCs as confirmed in the present study. Furthermore, there was no primary toxicity seen with DSCs doses up to 40 × 10^6^/kg.

One way of overcoming pulmonary trapping of cells and facilitate homing is to administer cells intra-arterially upstream of target tissue. In this study we found that intra-aortal injections of DSCs in rats does not cause animal morbidity or tissue injury and result in DSC distribution in the abdominal organs instead of the lungs.

In patients infused with I-111-marked DSCs, signals were first found in the lung and after 48 h in liver and spleen. This is in contrast to the present study in mice where Luciferase-marked DSCs were found in the lungs of the animals up to 4 days, but in no other organ (Figure 4). This difference may be due to that Luciferase is a marker for live cells, whereas I-111 will also appear on dying cells. This seems reasonable due to the xeno-reactive mice immune system, which may distract the human DSCs already in the lung.

In this study, treating animals with doses up to 40 × 10^6^/kg we did not see any abnormal changes in laboratory values, including lymphocytes, hemoglobin, antithrombin, coagulation parameters, haptoglobin, complement C3, albumin, creatinine, liver enzymes, triglycerides, cholesterol, and HDL-cholesterol. This confirms that even extremely high doses of DSCs seem safe in accordance with clinical safety studies using doses around 1 × 10^6^ DSCs/kg. Histology of all examined organs did not show any adverse effects by infusion of DSCs.

The results from the clotting experiments confirmed previous data (18). DSCs had stronger clotting than BM-MSCs. By the addition of low dose heparin, clotting was entirely abrogated (Figure 7). Kinetics for *in vivo* monitoring of systemic coagulation and complement activation markers using DSCs and MSCs infusion in mice, revealed a weak triggering of the coagulation and complement cascades. We found similarly to our results from *in vitro* blood exposure, that DSCs without heparin were slightly more pro-coagulant *in vivo* than BM-MSCs. These data support the use of heparin when infusing stromal cells to patients for clinical use. Heparin also prevented BM-MSCs induced coagulation and the acute adverse events in experimental colitis (27).

In the comparison between BM-MSCs and DSCs, the former donors were slightly older. BM-MSCs were taken from bone marrow transplant donors with an age ranging from 30 to 50 years of age. The DSCs were from fertile women ranging in age between 20 and 35 years of age. The difference in donor age, most probably had no effect on the differences seen with coagulation and other parameters. We previously found that donor age had no impact on GvHD response to MSC therapy (46). We also reported that DSCs were more effective to treat acute GvHD than BM-MSCs (29). The differences in efficacy are more likely due to differences in source of MSC, than to MSC donor age.

As outlined in the introduction, it was demonstrated in mice that BM-MSCs following contact with activated T cells or NK cells, activate caspase and undergo apoptosis, which appears to be critical for MSC-induced systemic immunosuppression *in vivo* (11). Apoptosis is induced by the bystander release of cytotoxic granules by the activated immune cells. The killing is contact-dependent but not antigen-specific and does not require the engagement of the immunological synapses. MSCs, which undergo apoptosis, promote the chemotaxis of monocytes and macrophages that phagocytose the MSCs (8, 12–14). By these mechanisms, the macrophages produce anti-inflammatory activities *in vivo*, which are dependent, among others, on host indoleamine deoxygenase. If similar mechanisms are also induced by DSCs remains to be elucidated.

The present study demonstrates the safety of infusing DSCs i.v. and i.a. with normal appearing in rats and mice, no side effects and no toxicity to any organs. Minor effects on coagulation were normalized by heparin infusion.

## Data Availability Statement

The raw data supporting the conclusions of this manuscript will be made available by the authors, without undue reservation, to any qualified researcher.

## Author Contributions

BS, GM, SH, and OR: concept and design. SG, GM, SH, and OR: financial support. BS and OR: administrative support. BS, GM, FA, ES, BK, RC, JK-M, SG, GM, SH, and OR: collection and assembly of data and final approval of manuscript. BS, GM, FA, GM, SH, and OR: data analysis and interpretation.

### Conflict of Interest

The authors declare that the research was conducted in the absence of any commercial or financial relationships that could be construed as a potential conflict of interest.
