# Supplementary material for: Preclinical Toxicity Evaluation of Clinical Grade Placenta-Derived Decidua Stromal Cells
Source: Front Immunol. 2019 Nov 19;10:2685. doi: 10.3389/fimmu.2019.02685 (PMC6877599; doi:10.3389/fimmu.2019.02685)
Supplement: Supplementary file 1 [file Data_Sheet_1.pdf]

SUPPLEMENTARY DATA

Table S1: Antibody panel used for flow cytometry

Antibody / Conjugate	Target Antigen / Fluorescent Conjugate	Host	Code #	Company	Target
IgG1/2a (FITC/PE)	Mouse IgG1/2 Isotype (FITC/PE-conjugated)	Mouse Monoclonal	#342409	Becton Dickinson	Isotype Control Antibody Label
IgG1/2a (PE/FITC)	Mouse IgG1/2 Isotype (PE/FITC-conjugated)	Mouse Monoclonal	#340394	Becton Dickinson	Isotype Control Antibody Label
CD45/14 (FITC/PE)	Anti-human CD45/14 (FITC/PE-conjugated)	Mouse Monoclonal	#342408	Becton Dickinson	Negative Control Markers on MSCs
CD31 (FITC)	Anti-human CD31 (FITC-conjugated)	Mouse Monoclonal	#303110	Bio Legend	Negative Control Marker on MSCs
CD73 (PE)	Anti-human Ecto-5’ Nucleotidase (PE)	Mouse Monoclonal	#550257	Becton Dickinson	Positive Control Marker on MSCs
CD90 (FITC)	Anti-human Thy-1 (FITC-conjugated)	Mouse Monoclonal	#555595	Becton Dickinson	Positive Control Marker on MSCs
CD105 (PE)	Anti-human Endoglin (PE-conjugated)	Mouse Monoclonal	#326040	Ancell	Positive Control Marker on MSCs
CD142 (PE)	Anti-human Tissue Factor (PE-conjugated)	Mouse Monoclonal	#550312	Becton Dickinson	Tissue Factor Antigen on MSCs
